# SNP discovery in swine by reduced representation and high throughput pyrosequencing

**DOI:** 10.1186/1471-2156-9-81

**Published:** 2008-12-04

**Authors:** Ralph T Wiedmann, Timothy PL Smith, Dan J Nonneman

**Affiliations:** 1 USDA, ARS, U.S. Meat Animal Research Center, State Spur 18D, P.O. Box 166, ay Center, NE 68933-0166, USA

## Abstract

**Background:**

Relatively little information is available for sequence variation in the pig. We previously used a combination of short read (25 base pair) high-throughput sequencing and reduced genomic representation to discover > 60,000 single nucleotide polymorphisms (SNP) in cattle, but the current lack of complete genome sequence limits this approach in swine. Longer-read pyrosequencing-based technologies have the potential to overcome this limitation by providing sufficient flanking sequence information for assay design. Swine SNP were discovered in the present study using a reduced representation of 450 base pair (bp) porcine genomic fragments (approximately 4% of the swine genome) prepared from a pool of 26 animals relevant to current pork production, and a GS-FLX instrument producing 240 bp reads.

**Results:**

Approximately 5 million sequence reads were collected and assembled into contigs having an overall observed depth of 7.65-fold coverage. The approximate minor allele frequency was estimated from the number of observations of the alternate alleles. The average coverage at the SNPs was 12.6-fold. This approach identified 115,572 SNPs in 47,830 contigs. Comparison to partial swine genome draft sequence indicated 49,879 SNP (43%) and 22,045 contigs (46%) mapped to a position on a sequenced pig chromosome and the distribution was essentially random. A sample of 176 putative SNPs was examined and 168 (95.5%) were confirmed to have segregating alleles; the correlation of the observed minor allele frequency (MAF) to that predicted from the sequence data was 0.58.

**Conclusion:**

The process was an efficient means to identify a large number of porcine SNP having high validation rate to be used in an ongoing international collaboration to produce a highly parallel genotyping assay for swine. By using a conservative approach, a robust group of SNPs were detected with greater confidence and relatively high MAF that should be suitable for genotyping in a wide variety of commercial populations.

## Background

The identification of genes and mutations that lead to genetic variation in complex, economically important traits in livestock has been hindered by the lack of genomic sequence, adequate map density and effective platforms for high density genotyping. It is estimated that linkage disequilibrium extends for hundreds of kilobases in the pig [1] and that 30,000–50,000 SNP would be necessary for whole genome associations in livestock [2,3]. The availability of livestock genome sequence, a high density of markers, and cost effective SNP genotyping will allow genome-wide association studies in swine. A major limitation to the development of highly parallel genotyping assays for swine is a lack of suitable SNPs for genotyping. To date there are a little over 8,400 SNPs for swine in dbSNP, but many of these are clustered into a small number of sequences that do not effectively cover the genome [4]. While many SNP will surely be discovered by genome sequencing, because of low sequence coverage the conversion rate of putative SNP and their minor allele frequency won't be known until tested across populations. In order to identify large numbers of randomly distributed SNPs for swine, we chose to construct a reduced representation library (RRL) to reduce the complexity of the genome and to use massively parallel second-generation sequencing to identify large numbers of high-confidence SNP for high density genotyping on a cost effective platform.

Reduced representation was first used to construct an SNP map in human to scan the genome for haplotypes associated with disease [5]. Recently, reduced representation was coupled with second-generation sequencing technology for SNP detection, estimation of allele frequency and validation in cattle [6]. Reduced representation sequencing has also been used successfully for gene discovery [7], methylation analysis [8] and genomic characterization of repetitive genomes [9]. Because reduced representation reduces the complexity of the genome being sampled by orders of magnitude, doesn't require prior knowledge of genome sequence, and samples identical regions from different individuals dispersed across the genome, it is an ideal strategy for SNP discovery in species without a complete genome sequence.

## Results

### Reduced Representation Library Selection

Six enzymes were screened for suitability for RRL construction, with the goal of minimizing repetitive content in the target size range of 450 bp. For comparison, we also digested bovine DNA with which we had previous experience [6]. In contrast to the result with bovine DNA, porcine digests displayed no or few bands on the gel (Additional file 1), suggesting that the most abundant repetitive elements in swine do not contain multiple restriction enzyme sites. Unfortunately, this has the effect of making it more difficult to avoid repetitive elements in RRL construction, so we decided to empirically test the enzymes to identify the enzyme producing the lowest percentage of fragments containing repeat elements. Initial test sequencing of libraries constructed from different enzymes revealed that the repetitive DNA content was similar for most enzymes (Table 1). This result was supported by what was observed on the gel, i.e., that the repetitive DNA did not concentrate in bands and was dispersed throughout the libraries (Additional file 1). The *Bst*UI library contained the least repetitive DNA detected by RepeatMasker, but after aligning individual contigs to pig genomic sequence it was found to contain a high proportion (25%) of centromeric repeats. Because of the probability of a high number of unmapped SNPs locating to centromeric regions, this library was not pursued. The *Hae*III library was chosen for pyrosequencing because of the large number of predicted fragments and the relatively lower amount of repetitive DNA. Also, *in silico *digestion of the draft sequence of pig chromosome 1 indicated there was not a concentration of repetitive bands present in the targeted region around 450 base pair (Additional files 2 and 3).

**Table 1:** Percent repetitive element content of reduced representation libraries.

Library^1^	Restriction Enzyme	Site	SINEs^2^	LINEs	LTR	DNA	Total	Centromeric Repeats^3^
GSS	Sheared	random	11.30	16.14	2.80	1.51	31.75	0.00
pRRL1	*Alu *I	AG|CT	10.21	15.63	2.46	3.16	31.46	0.00
pRRL2	*Hae *III	GG|CC	13.19	11.33	3.11	1.79	29.42	0.00
pRRL3	*Bst*U I	CG|CG	1.98	0.40	0.03	0.42	2.83	23.83
pRRL4	*Dra *I	TTT|AAA	32.04	4.85	1.67	1.09	39.65	0.00
pRRL5	*Pvu *II	CAG|CTG	20.99	4.91	1.71	1.25	28.86	6.18

^1 ^GSS refers to genome survey sequence and values were obtained from Wernersson et al., 2005 [14].

^2 ^Values are given as percent of total. SINEs refers to short interspersed elements porcine SINE elements and MIRs; LINEs refers to LINE1, LINE 2, L3/CR1 and RTE elements; LTR refers to long terminal repeats MaLRs, and ERVL; DNA refers to MER1 and MER2-type elements. Total refers to percent of repetitive content identified by RepeatMasker.

^3^Values in the centromeric repeat column represent percent of sequences that contain a centromeric repeat and not percent of sequence content.

### GS FLX Sequencing and Assembly

A total of 5,024,039 reads were obtained in 11 runs of the GS FLX instrument producing 1,167,904,923 total bases of sequence (average read length of 232 bp), with 87% of bases having a quality score of 20 or more. Most of the reads (96.4%) started with "CC", as expected for these restriction enzyme digested fragments. Less than 1% of reads had an internal *Hae*III recognition site (GGCC), indicating that digestion was complete. About 32.6% of the sequence was found to be repetitive DNA by RepeatMasker, consistent with the results obtained from the Sanger sequenced *Hae*III fragments (see Table 1). The percentage of bases called as "N" was 0.02%.

**Figure 1:**
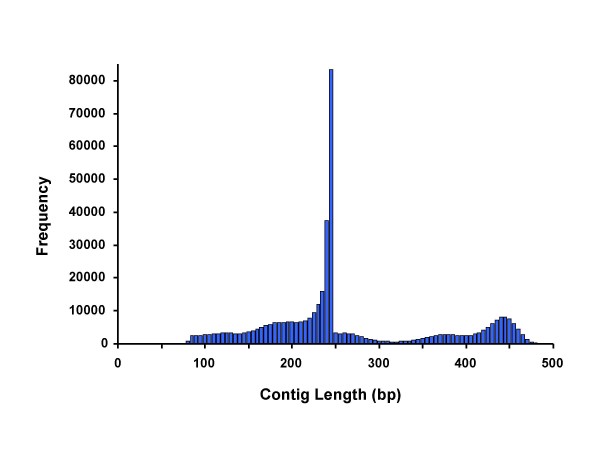
**Distribution of the contig lengths showing that most of the contigs consist of reads from one end of the restriction fragments.** About 25% of the contigs span the entire restriction fragment.

The Newbler assembler, version 1.1.03, assembled 73% of the unmasked reads into 421,060 contigs, which were used to define the reference sequence for SNP discovery. Attempts to increase the number of reads in the assembly resulted in fatal software errors. Although 27% of the original reads were not used in the assembly, they were included in the mapping and SNP detection steps. Figure 1 shows the profile of contig lengths, indicating that most of the contigs were the length of a single read, but about 100,000 contigs were longer as reads from opposite directions overlapped in the middle to fully cover the library fragments. The total length of the 421,060 contigs was 110,823,689 bp indicating an average unmasked read coverage of 7.65×. Although N's were quite rare in the reads, 4.8% of the bases called by the assembler were "N", mostly concentrated in a small fraction of the contigs. Over 70% of the contigs were free of N's and 78% had less than 1% N content. The contig sequences are available in dbSTS [GenBank: BV729586 to BV999999, GF000001 to GF089508 and GF089703 to GF091743]. The SNPs are available in dbSNP [GenBank: ss107796326 to ss107911925].

### SNP Detection

Prior to the SNP discovery step, the repetitive content of the reads was masked by RepeatMasker and only those reads containing a 50 bp length of non-repetitive sequence were retained. Repetitive sequence near the ends of the reads was trimmed. Using the Newbler unmasked assembly (421,060 contigs) as the reference, the ssaha2 software mapped 2,189,534 (72.0%) of the 3,041,168 repeat-masked and trimmed reads to the reference contigs, placing a total of 468,787,385 bp onto the reference sequence. The repetitive regions of the reference assembly are not expected to have coverage from the repeat-masked reads, and, in fact, 27% of the reference had no reads mapped onto it. For the positions that did have coverage, the average depth was 5.84×. We used ssaha_pileup to detect variation between the mapped reads and the reference, and among the mapped reads.

**Figure 2:**
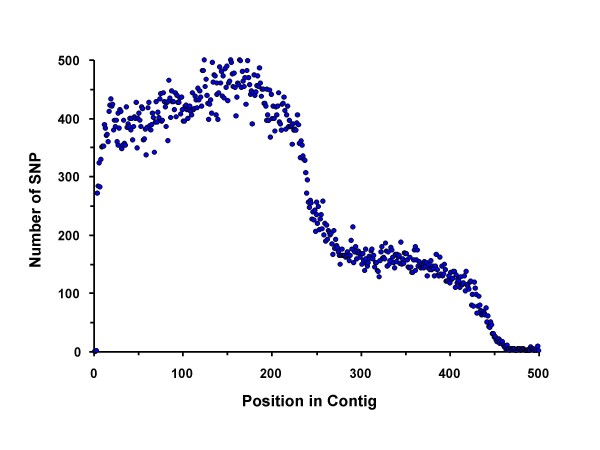
**The plot shows the distribution of 115,572 SNPs by position in the contigs.** The number of SNPs mirrors the profile of contig lengths.

**Figure 3:**
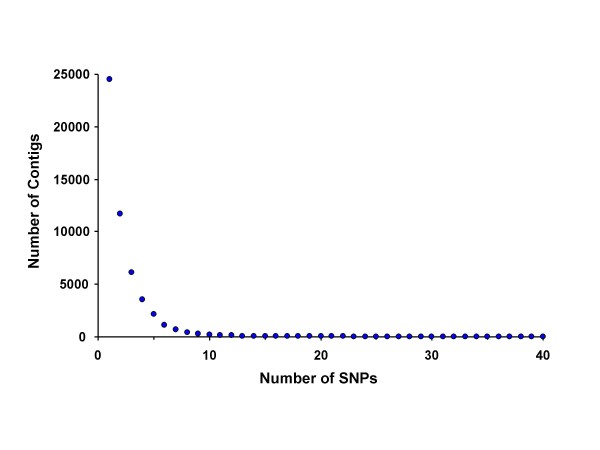
The distribution of contigs by number of SNPs.

**Figure 4:**
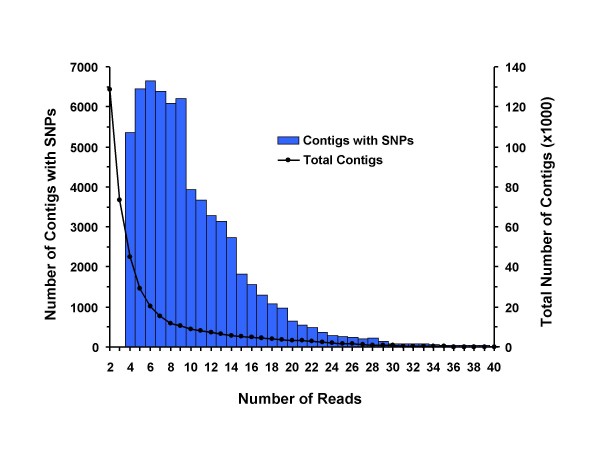
**Comparison of the distribution of total contigs to contigs containing SNPs as the depth of coverage changes.** The average depth of coverage where SNP were detected was 12.6.

Looking only at the SNP in the masked reads, we filtered out those that had a gap, an N or a third allele. We also required that both alleles were observed at least twice. We did not filter based on quality scores or on the presence of nearby homopolymers, as has been done with 454 data [10,11]. This approach identified 115,572 SNPs in 47,830 unique contigs (11.4% of the 421,060 total contigs) (Figure 2). The SNPs were distributed throughout the contigs in proportion to the sequence depth and contig length across the contigs, i.e., more SNPs were found in shorter contigs than in contigs that were assembled with reads from both ends of the 450 bp fragments (Figure 2). Nearly half (47%) of all contigs contained a single SNP (Figure 3). Most of the SNP-containing contigs were produced from 5–9 reads, but the average coverage at the positions of the SNPs is 12.6 as a result of our requirement for two occurrences of the minor allele (Figure 4). Including the repetitive content of the reference contigs, we detected 1 non-repetitive SNP per 959 bp of reduced representation sequence. Looking only where the reference contigs had a minimum coverage depth of 4-fold, the rate of SNP detection is 1 SNP per 370 bp (115,572 SNP in 42,827,328 bp). Because the observed rate of SNP detection as a function of depth continues to increase as the depth increases, we would expect an even higher rate of SNP discovery with deeper sequencing of the RRL.

**Figure 5:**
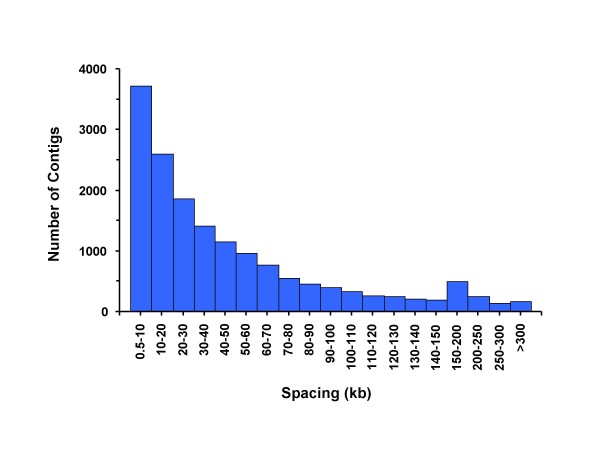
Contig spacing along the sequenced pig chromosomes 1, 4, 5, 7, 11, 13 and 14.

The average predicted MAF of SNPs discovered in the contigs with a minimum of four reads was 0.356, which is likely to be an overestimate since SNP with relatively low coverage are not accurately estimated and tend toward a 50% MAF due to the selection criteria. Forty-six percent of the contigs containing SNPs (22,045) mapped to the finished pig genomic sequence  with an average spacing of 44.2 kb and a median spacing of 29.3 kb (Figure 5).

### SNP Genotyping and Validation

**Figure 6:**
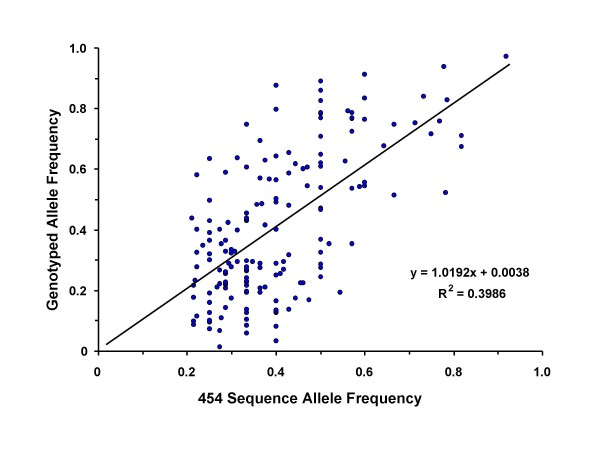
Correlation between allele frequency estimated by 454 sequencing and genotyping.

Multiplex assays targeting 192 putative SNP randomly selected from the set of SNP mapping to finished porcine genomic sequence were attempted in order to estimate the conversion rate and compare the predicted MAF to an empirical MAF derived from genotyping a sample of 192 animals representing 12 breeds. Nine SNP failed as a result of the assay design (unrecognized SNPs under probe or primers), and seven failed to produce an amplification product when combined in the multiplex, leaving 176 SNP for analysis. From this reduced set, eight were monomorphic indicating an overall conversion rate of 95.5% (168 of 176). Of the eight SNP assays that were monomorphic, four contained homopolymers of 4–9 nucleotides flanking the polymorphic site and one contained a short dinucleotide repeat. Two monomorphic SNPs were in contigs that contained only four reads. Fifteen successful assays contained homopolymers of 5 or more nucleotides flanking the SNP. The SNP position in the contig and predicted minor allele frequency did not seem to affect the validity of the SNP (data not shown). The MAF for those SNP genotyped on the Sequenom platform averaged 0.28 and the average heterozygosity was 0.27. The MAF and heterozygosity for individual breeds in the panel are shown in Table 2. The minor allele frequencies were similar between breeds, except for the outgroups (Meishan and European Wild Boar) not included in the discovery pool, which were lower. Fewer uninformative SNPs were found in Landrace and Yorkshire compared to other breeds (Table 2.) The correlation between marker allele frequencies determined by sequencing and genotyping was 0.58 in the animals represented in the discovery pool and 0.63 in the 192 animal breed panel (Figure 6), similar to that reported for a bovine RRL sequenced to 20-fold average coverage [6] though somewhat lower, probably a result of lower coverage depth reducing estimation accuracy.

**Table 2:** Average minor allele frequencies by breed for 168 sampled SNPs.

Breed	Number of Animals^1^	Number in Pool^2^	Genotyped (%)^3^	Monomorphic (%)^4^	Average MAF	Heterozygosity
Berkshire	7	3	98.21	20.83	0.2142	0.2866
Duroc	40	4	98.13	15.48	0.2003	0.2537
Eur. Wild Boar	5	0	99.28	44.64	0.1533	0.2520
Hampshire	29	4	98.81	12.50	0.2131	0.2692
Landrace	43	4	98.01	3.57	0.2457	0.3017
Meishan	5	0	98.10	44.64	0.1447	0.1881
Pietrain	6	2	98.81	25.00	0.2041	0.2892
Yorkshire	41	4	98.88	5.36	0.2429	0.2887

^1^Number of animals of each breed genotyped.

^2^Number of animals of each breed in the discovery library.

^3^Percent of animals that produced a genotype call.

^4^Percent of markers that were monomorphic for that breed.

## Discussion

These results indicate that reduced representation sequencing coupled with second-generation sequencing technology provides the detection of a large number of valid SNPs at a much lower investment of time and expense than conventional methods. This approach was recently used to discover a large number of SNP in cattle [6] by deep-sequencing RRLs to simultaneously identify and determine MAF of SNP using short reads that were mapped to the bovine genome. This study differs by the use of longer sequence reads, which are more likely to map to the unfinished pig genome sequence or that of related species, provide the ability to design assay primers for many different genotyping platforms and allow the detection of neighboring SNP in the same fragment. Although the rate of detection per base was lower than our initial prediction based on Sanger sequencing of PCR products from pig genomic DNA [4], the conservative approach of requiring that the variation be seen at least twice to be called polymorphic resulted in a large number of accurately identified SNPs as reflected by the high success rate in validation with a false discovery rate of less than 5%. The success rate could probably be further improved by eliminating SNPs residing in homopolymeric regions, because the pyrosequencing method has difficulty with homopolymers and thus the error rate of sequencing versus actual SNP is increased. Additional sequencing of the *Hae*III RRL library to get deeper coverage would likely uncover more SNPs, but would bias toward those with lower minor allele frequencies. From this resource, assays could be designed from the greater than 50,000 non-repetitive loci (contigs) for high-density genotyping providing a reasonable distribution of markers across the genome. Although map positions will only be known for those contigs that fall within the sequenced regions of the pig genome, the remaining contig positions should be available in the near future. Alternatively, the contigs could be mapped by linkage or with a high resolution radiation hybrid panel [12], or by similarity to the completely sequenced human or bovine genomes.

## Conclusion

Although SNP are abundant in the genome and are amenable to high-throughput genotyping technology, the identification of a large number of informative SNP spaced over the genome suitable for whole genome association is a difficult and expensive task. The combination of next-generation sequencing technology and reduced representation of pooled genomes provides a powerful and efficient strategy to discover large numbers of genetic markers in a target population. The approach to sample several unrelated animals of different breeds and sequence to sufficient depth for reliable SNP identification allowed the ability to detect many common SNP present at a high MAF. The SNPs identified in this report will provide a much needed resource for genetic studies in swine and will contribute to the development of a high density, cost effective genotyping platform for swine.

## Methods

### Design and Construction of Reduced Representation Libraries

The libraries were designed to generate fragments with a typical length of 450 basepair to allow for a 50 bp overlap of reads from opposite fragment ends assuming a 250 bp median read length from the GS FLX instrument (454 Life Sciences, Branford, CT, USA). Genomic DNA was extracted from semen of 21 unrelated boars (International Boar Semen, Eldora, IA) representing the seven most predominant industry breeds (four each of Duroc, Landrace, Yorkshire, Large White and Hampshire breeds, three Berkshire and two Pietrain) and from five Duroc-Landrace-Yorkshire cross-bred boars from the United States Meat Animal Research Center (USMARC) resource population. Equal amounts (40 μg) of DNA were pooled and digested overnight to insure complete digestion with 5 U/μg of *Alu*I, *Bst*UI, *Dra*I, *Hae*III, and *Pvu*II (New England Biolabs, Beverly, MA, USA) to produce 5 fragment libraries. Each of these enzymes produces fragments with blunt ends, which improves ligation efficiency of adaptors in the preparation of single strand libraries for sequencing on the 454 platform (T. Smith, unpublished data). Digested genomic DNA was fractionated in a 5% polyacrylamide gel immobilized to GelBond film (FMC, Rockland, ME, USA), stained with CyberGold (Molecular Probes, Eugene, OR, USA), visualized on a DarkReader (Clare Chemical Research, Dolores, CO, USA; see Additional file 1), and a gel section containing digested fragments of approximately 450 bp (427–456 bp) was removed. The gel sections were crushed by centrifugation through an 18-gauge needle hole in a microfuge tube and DNA was eluted from the gel pieces by incubation at 37°C overnight in 0.5 M ammonium acetate and 0.1 mM EDTA. To evaluate each enzyme for suitability in large-scale sequencing, samples of fragments generated by each enzyme were cloned into pBluescript, and two 384-well plates of transformed clones from each library were sequenced on an ABI 3730 (Applied Biosystems, Foster City, CA, USA) The repetitive DNA content of the libraries was determined using RepeatMasker  after running Crossmatch  to remove vector sequence.

### DNA Pyrosequencing

A single strand library was prepared from the *Hae*III fragments for sequencing on the GS FLX platform as recommended by the manufacturer (454 Life Sciences Corporation, Branford, CT, USA). Six micro-bead sequencing runs of the *Hae*III library were performed as a service by 454 Life Sciences Corporation (Branford, CT, USA) using a Roche-454 GS FLX sequencer. Five additional machine runs were performed on a Roche-454 GS FLX sequencer at USMARC.

### Assembly of the reference sequence and SNP Detection

To generate the reference contigs used for SNP discovery, unmasked sequence reads were assembled using the Newbler algorithm (version 1.1.03) provided with the GS FLX sequencer. Repeat-masked reads were removed from further analysis if they contained less than 50 consecutive non-masked bases in one region leaving 60% of the initial reads. These reads were trimmed of repetitive sequence near their ends prior to either assembly or mapping. The RepeatMasked reads were then mapped onto the reference contigs using ssaha2 software and SNP were detected by ssaha_pileup [13]. Putative SNPs were tagged if each of two alleles appeared at least twice and no other alleles were detected; therefore a minimum depth of four reads was necessary in a contig to detect SNPs.

### Validation of SNPs

A group of 1,000 SNPs was randomly selected from among those that mapped to the finished pig chromosome sequence that represented differing allele frequencies, positions in the contig and depth of reads. An excess of SNPs was selected to allow for multiple SNPs in a contig and to provide enough targets for multiplex assay design. For contigs containing more than one SNP, only one SNP was selected per contig for validation. In addition, SNP were discarded if the contig did not have at least 30 bp on either side of the SNP to allow for amplification primer design. Multiplex assays were designed for the Sequenom MASSARRAY^® ^system using the MASSARRAY^® ^Assay Design software (Sequenom, San Diego, CA, USA). Assays were designed for 192 unique SNP with thirty-two SNPs in each of six multiplexes. Each amplification primer had a 10-base tag added to ensure that the amplification primer masses were outside the range of the allele masses and amplicon lengths with tags were approximately 90 bp. Reaction conditions were performed by iPLEX™ chemistry as recommended by Sequenom. The SNP were genotyped across a panel of 192 animals which included the 21 discovery animals and consisted of about 40 animals each for the Duroc, Landrace and Yorkshire breeds, 29 Hampshire, and 2–7 animals each for the Berkshire, Chester White, European Wild Boar, Fengjing, Meishan, Minzhu, Pietrain, Poland China, and Spotted breeds

## Authors' contributions

TPLS and DJN designed the RRL strategy, DJN constructed the RRL and validated SNPs, TPLS directed sequencing and RTW performed the sequence analysis and bioinformatics. All authors read and approved the final manuscript.

## Additional data files and information

The sequence and nucleotide variation has been submitted to GenBank dbSTS and dbSNP databases. The Accession Numbers are: [GenBank: BV729586 to BV999999, GF000001 to GF089508 and GF089703 to GF091743]. The SNPs are submitted under the handle MARC in batch number 2008-11-06 [GenBank: ss107796326 to ss107911925].

Additional data is included in the form of three figures (Additional files 1, 2 and 3).

## Supplementary Material

Additional file 1 **Polyacrylamide gel of restriction digests of bovine and porcine DNA.** Two micrograms of genomic DNA was digested with the indicated enzyme and electrophoreses in 5% polyacrylamide gel. Repetitive elements are clearly seen as bands in the gel. The numbers to the left of the fragment size markers have units of bp. Click here for file

Additional file 2 **Size distribution of restriction fragments from an *in silico Hae*III digest of pig chromosome 1.** Click here for file

Additional file 3 **Total content of DNA by fragment size in the *in silico *digest of chromosome 1.** Repetitive elements are seen as spikes in the histogram. Click here for file
