# Supplementary figures and images for: SNP discovery in swine by reduced representation and high throughput pyrosequencing
Source: BMC Genet. 2008 Dec 4;9:81. doi: 10.1186/1471-2156-9-81 (PMC2612698; doi:10.1186/1471-2156-9-81)

*Alu* I    *Dra* I    *Hae* III    *Pvu* II

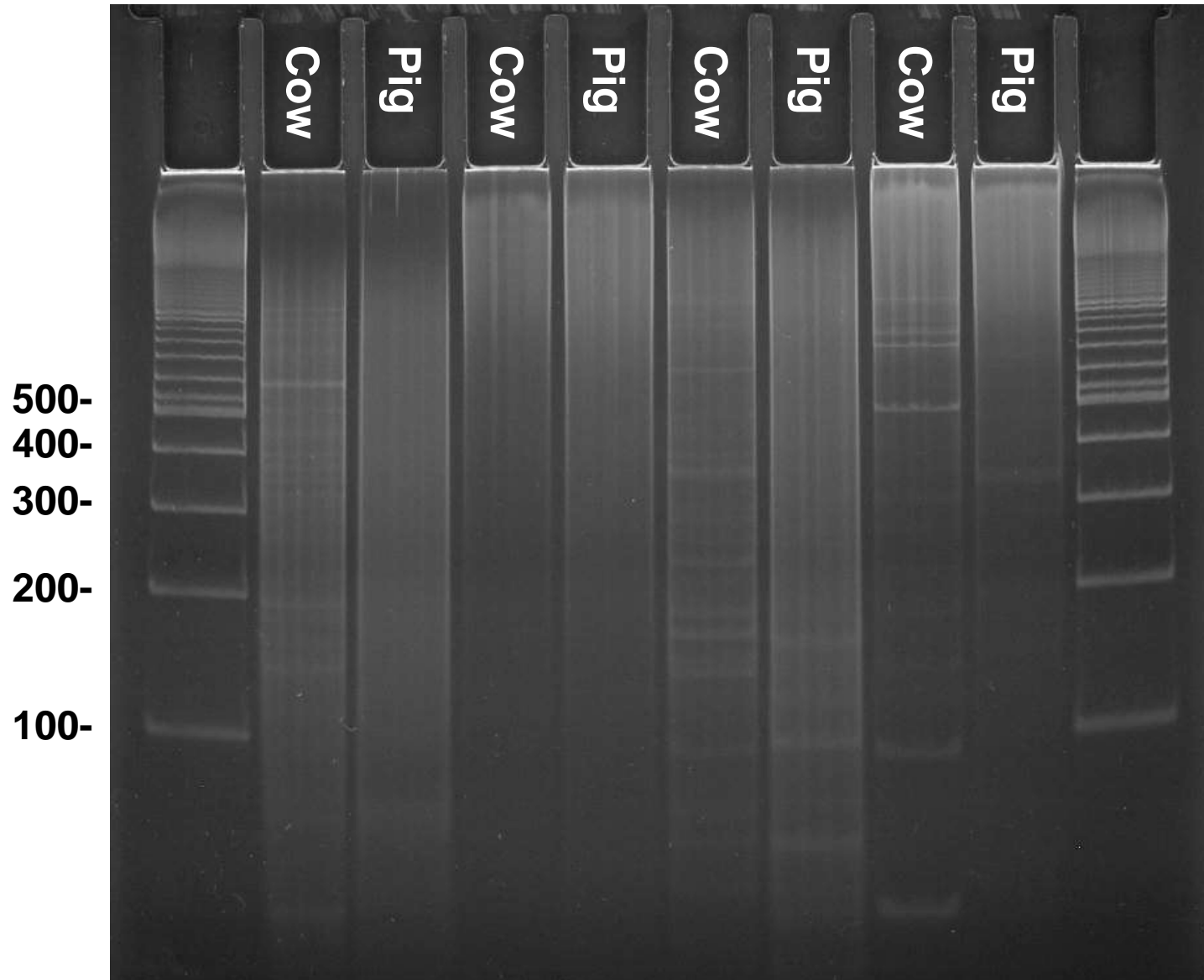

Supplement: Additional file 1 — Polyacrylamide gel of restriction digests of bovine and porcine DNA. Two micrograms of genomic DNA was digested with the indicated enzyme and electrophoreses in 5% polyacrylamide gel. Repetitive elements are clearly seen as bands in the gel. The numbers to the left of the fragment size markers have units of bp. [file 1471-2156-9-81-S1.pdf]

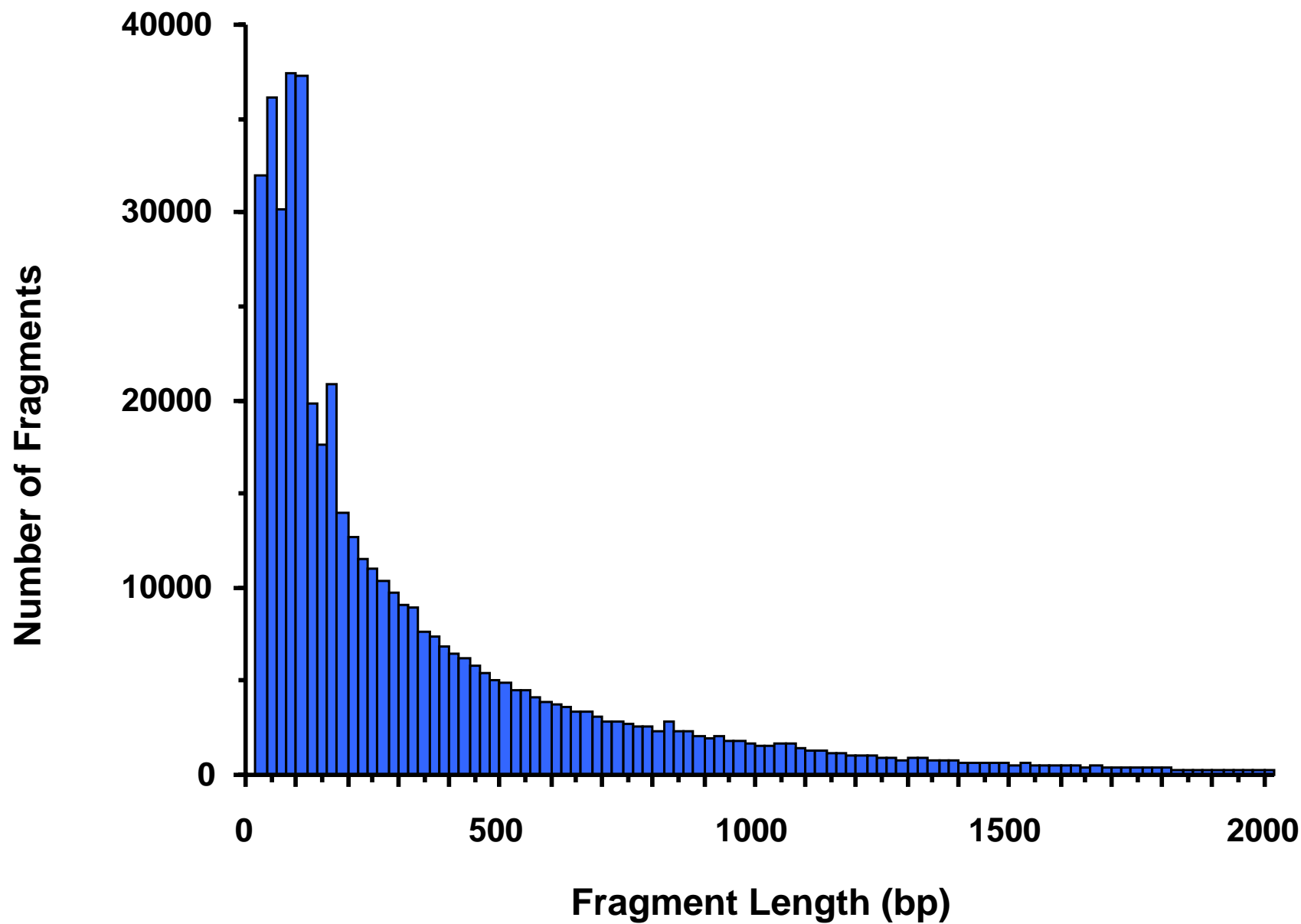

Supplement: Additional file 2 — Size distribution of restriction fragments from an in silico HaeIII digest of pig chromosome 1. [file 1471-2156-9-81-S2.pdf]

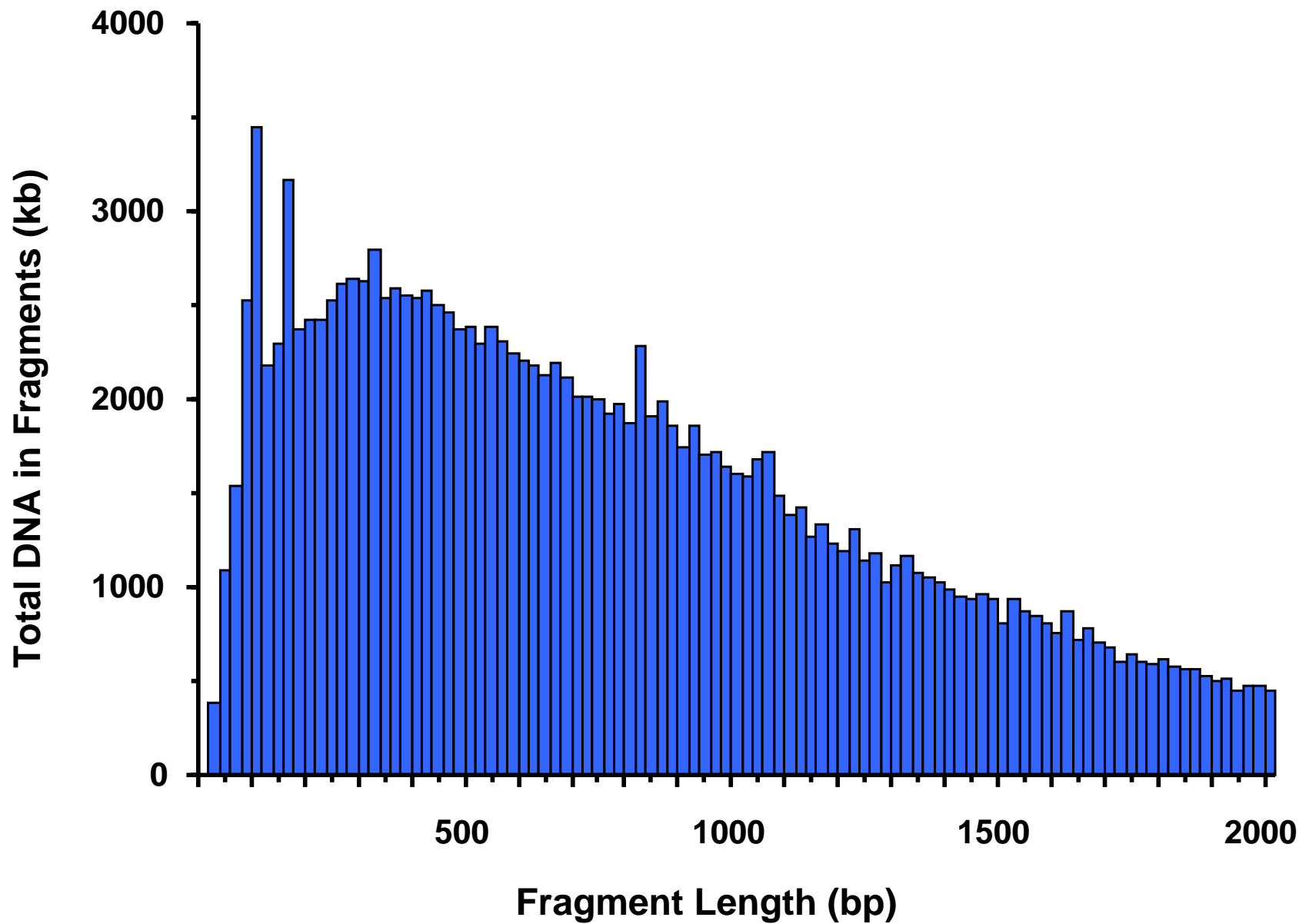

Supplement: Additional file 3 — Total content of DNA by fragment size in the in silico digest of chromosome 1. Repetitive elements are seen as spikes in the histogram. [file 1471-2156-9-81-S3.pdf]
